# Supplementary material for: Complex‐centric proteome profiling by SEC‐SWATH‐MS
Source: Mol Syst Biol. 2019 Jan 14;15(1):e8438. doi: 10.15252/msb.20188438 (PMC6346213; doi:10.15252/msb.20188438)
Supplement: Supplementary file 8 — Dataset EV7 [file MSB-15-e8438-s008.zip › feature_plots_string/P62072.pdf]

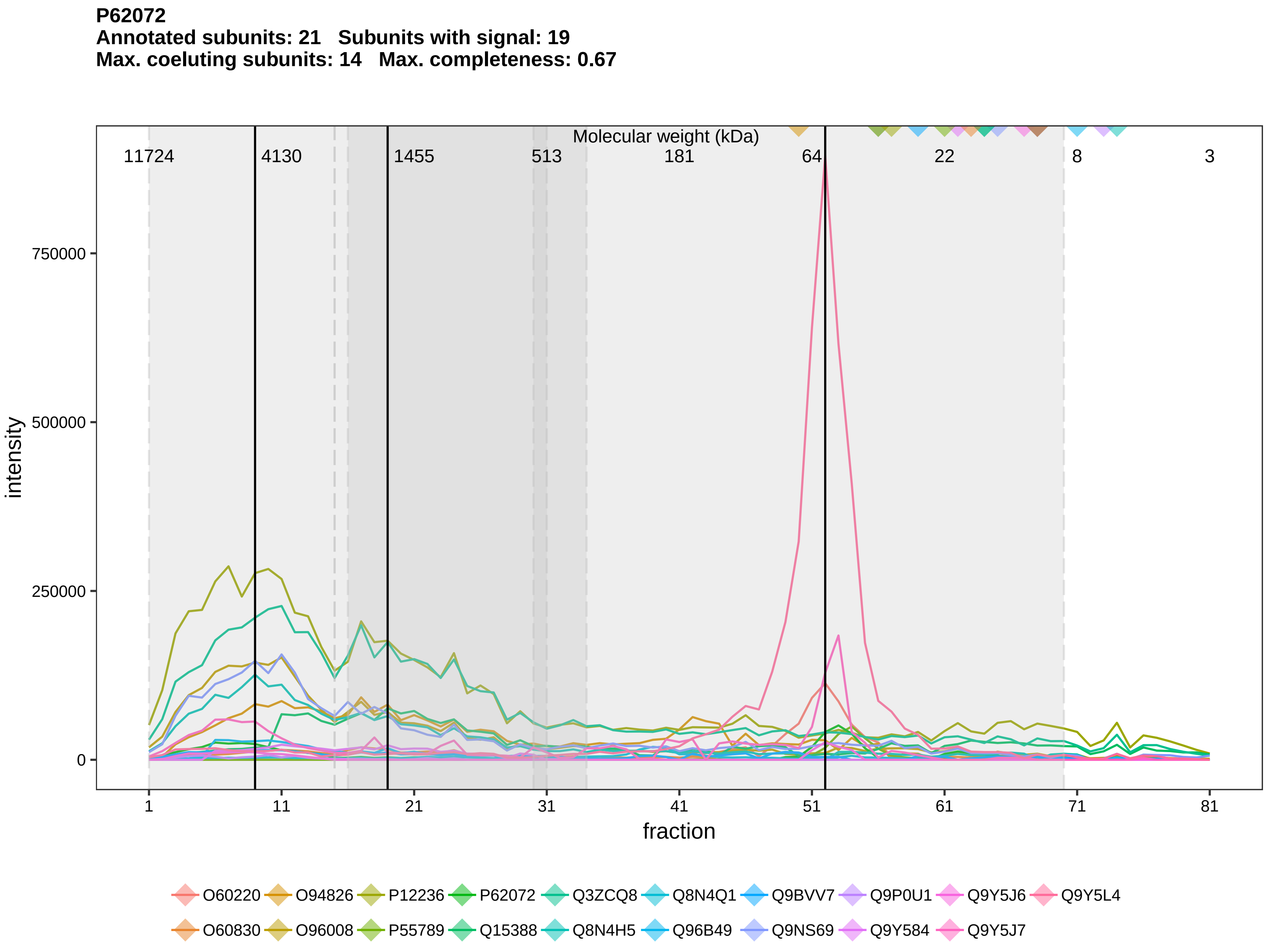
P62072
Annotated subunits: 21 Subunits with signal: 19
Max. coeluting subunits: 14 Max. completeness: 0.67
Molecular weight (kDa)
11724
4130
1455
513
181
64
22
8
3
intensity
750000
500000
250000
0
fraction
1
11
21
31
41
51
61
71
81
O60220
O94826
P12236
P62072
Q3ZCQ8
Q8N4Q1
Q9BVV7
Q9P0U1
Q9Y5J6
Q9Y5L4
O60830
O96008
P55789
Q15388
Q8N4H5
Q96B49
Q9NS69
Q9Y584
Q9Y5J7
